# Combination of nanoparticle-based therapeutic vaccination and transient ablation of regulatory T cells enhances anti-viral immunity during chronic retroviral infection

**DOI:** 10.1186/s12977-016-0258-9

**Published:** 2016-04-14

**Authors:** Torben Knuschke, Olga Rotan, Wibke Bayer, Viktoriya Sokolova, Wiebke Hansen, Tim Sparwasser, Ulf Dittmer, Matthias Epple, Jan Buer, Astrid M. Westendorf

**Affiliations:** Institute of Medical Microbiology, University Hospital Essen, University of Duisburg-Essen, 45122 Essen, Germany; Institute of Inorganic Chemistry and Center for Nanointegration (CeNIDE), University of Duisburg-Essen, 45141 Essen, Germany; Institute of Virology, University Hospital Essen, University of Duisburg-Essen, 45122 Essen, Germany; Institute of Infection Immunology, TWINCORE, Centre for Experimental and Clinical Medicine, 30625 Hannover, Germany

**Keywords:** Chronic retrovirus infection, Nanoparticles-based vaccine, Regulatory T cells

## Abstract

**Background:**

Regulatory T cells (Tregs) have been shown to limit anti-viral immunity during chronic retroviral infection and to restrict vaccine-induced T cell responses. The objective of the study was to assess whether a combinational therapy of nanoparticle-based therapeutic vaccination and concomitant transient ablation of Tregs augments anti-viral immunity and improves virus control in chronically retrovirus-infected mice. Therefore, chronically Friend retrovirus (FV)-infected mice were immunized with calcium phosphate (CaP) nanoparticles functionalized with TLR9 ligand CpG and CD8^+^ or CD4^+^ T cell epitope peptides (GagL_85–93_ or Env gp70_123–141_) of FV. In addition, Tregs were ablated during the immunization process. Reactivation of CD4^+^ and CD8^+^ effector T cells was analysed and the viral loads were determined.

**Results:**

Therapeutic vaccination of chronically FV-infected mice with functionalized CaP nanoparticles transiently reactivated cytotoxic CD8^+^ T cells and significantly reduced the viral loads. Transient ablation of Tregs during nanoparticle-based therapeutic vaccination strongly enhanced anti-viral immunity and further decreased viral burden.

**Conclusion:**

Our data illustrate a crucial role for CD4^+^ Foxp3^+^ Tregs in the suppression of anti-viral T cell responses during therapeutic vaccination against chronic retroviral infection. Thus, the combination of transient Treg ablation and therapeutic nanoparticle-based vaccination confers robust and sustained anti-viral immunity.

**Electronic supplementary material:**

The online version of this article (doi:10.1186/s12977-016-0258-9) contains supplementary material, which is available to authorized users.

## Background

Effector T cells are crucial for the elimination of intracellular pathogens. Especially cytotoxic CD8^+^ T cells are predominantly important for the control of virus infections, i.e. human immunodeficiency virus (HIV) [1] or hepatitis C virus (HCV) [2, 3]. However, several evasion mechanisms of viruses, like viral evolution or exhaustion of effector T cells can contribute to persistent infection. Important inducer of dysfunctional CD8^+^ T cells are virus-induced or virus-expanded regulatory T cells (Tregs). Tregs belong to a specialized T cell subset which is characterized by the expression of the transcription factor Foxp3 and unique suppressive capacities that can limit anti-viral immune responses [4–6]. It was demonstrated that the low responsiveness and reduced proliferation of virus-specific T cells during chronic viral infection is associated with the expansion of Tregs which also affects the cytotoxic activity of virus-specific CD8^+^ T cells [6, 7]. Immune-based therapies have been considered as potent approach to efficiently reactivate impaired anti-viral effector immunity to control chronic viral infection. In this concern, depletion of Tregs during acute and chronic murine retroviral infection was shown to restore the functions of CD8^+^ T cells and to decrease viral loads [5, 6, 8, 9]. The results suggest that Treg-mediated immunosuppression is a significant factor in the maintenance of chronic viral infections, and that Treg-targeted immunotherapy could be a valuable component in therapeutic strategies to treat chronic retroviral infections.

Additionally, promising trials to efficiently reactivate CD8^+^ T cell responses have been made by confronting patient-derived dendritic cells (DCs) with HIV-specific antigens to induce virus-specific immune responses [10, 11] or by directly targeting lymph nodes with peptide-based vaccine vehicles [12]. In this context, nanoparticulate structures are discussed to be ideal vaccine delivery vehicles [13]. Inorganic calcium phosphate (CaP) nanoparticles are promising candidates for this purpose. Due to their small size (~100 nm) and their non-toxicity CaP nanoparticles feature ideal properties as delivery vehicles across cell membranes [14, 15]. After functionalization with TLR ligands such as CpG or poly(I:C), CaP nanoparticles efficiently activate cells of the innate and adaptive immune system [14]. In a recent study, we have shown that prophylactic immunization of mice with CaP nanoparticles, functionalized with virus-derived T cell epitopes and the TLR9 ligand CpG, leads to an activation of DCs and in consequence induced strong antigen-specific CD4^+^ and CD8^+^ T cell response. Of note, this vaccination strategy resulted in a powerful protection against influenza virus and Friend retrovirus (FV) infection [16, 17]. In addition, therapeutic vaccination of chronically FV-infected mice efficiently reactivated effector T cells which led to a significant decrease in viral loads.

FV is an immunosuppressive retrovirus complex of the non-pathogenic Friend murine leukemia virus (F-MuLV) and the pathogenic, replication defective spleen focus forming virus (SFFV) [18]. Disease-resistant mouse strains can control acute infection, but develop a life-long chronic infection. This is associated with the expansion and activation of Tregs [19], which down-regulate virus-specific cytotoxic CD8^+^ T cell activity [6], a conspicuous feature which in fact was also seen in HIV infection [20]. As no appropriate mouse model for studying HIV infection exists, the well-described FV infection model has been accepted to be suitable to understand basic concepts in retroviral immunity and to test novel vaccination strategies [21–24].

In this study, we aimed to identify whether nanoparticle-based vaccination and concomitant modulation of inhibitory Treg responses in chronically retrovirus infected mice results in potent anti-viral immunity and improves viral control. To our knowledge, we show for the first time that the combinational immune-based therapy of CaP nanoparticle vaccination and Treg ablation efficiently reactivates the cytotoxic potential of CD8^+^ effector T cells and leads to a dramatic drop in viral loads in chronic retroviral infection, stronger than each therapeutic treatment regimen alone. Thus, vaccine-induced reactivation of effector T cells during chronic viral infection is feasible but is strikingly enhanced by simultaneous immune modulation.

## Results

### Therapeutic vaccination of chronically infected mice with functionalized CaP nanoparticles transiently reactivates cytotoxic CD8^+^ T cells and reduce viral loads

Recently, we introduced functionalized CaP nanoparticles as excellent vaccination delivery system for the treatment of chronic retroviral infection [17]. Functionalized triple-shell CaP nanoparticles were prepared by subsequent precipitation and functionalization steps as described earlier [14, 16]. In brief, CaP solid cores were loaded with CpG and CD8^+^ or CD4^+^ T cell epitope peptides (GagL_85–93_ or Env gp70_123–141_) of FV and finally coated with a second layer of CaP and an outer shell of CpG for colloidal stabilization (Additional file 1: Figure S1A). Vaccination of mice with functionalized CaP nanoparticles efficiently activates the major DC subsets, therefore mediating ideal requirements for the initiation of adaptive immune responses [14, 16]. One shot-immunization of chronically FV infected mice with functionalized CaP nanoparticles reactivated virus-specific T cell responses accompanied by a significant decrease in viral loads with striking advantage over vaccination with soluble CpG ligand and viral peptides [17] (Additional file 1: Figure S1B–E).

### Foxp3^+^ CD4^+^ regulatory T cells down-regulate T cell responses after CaP nanoparticle vaccination

**Fig. 1:**
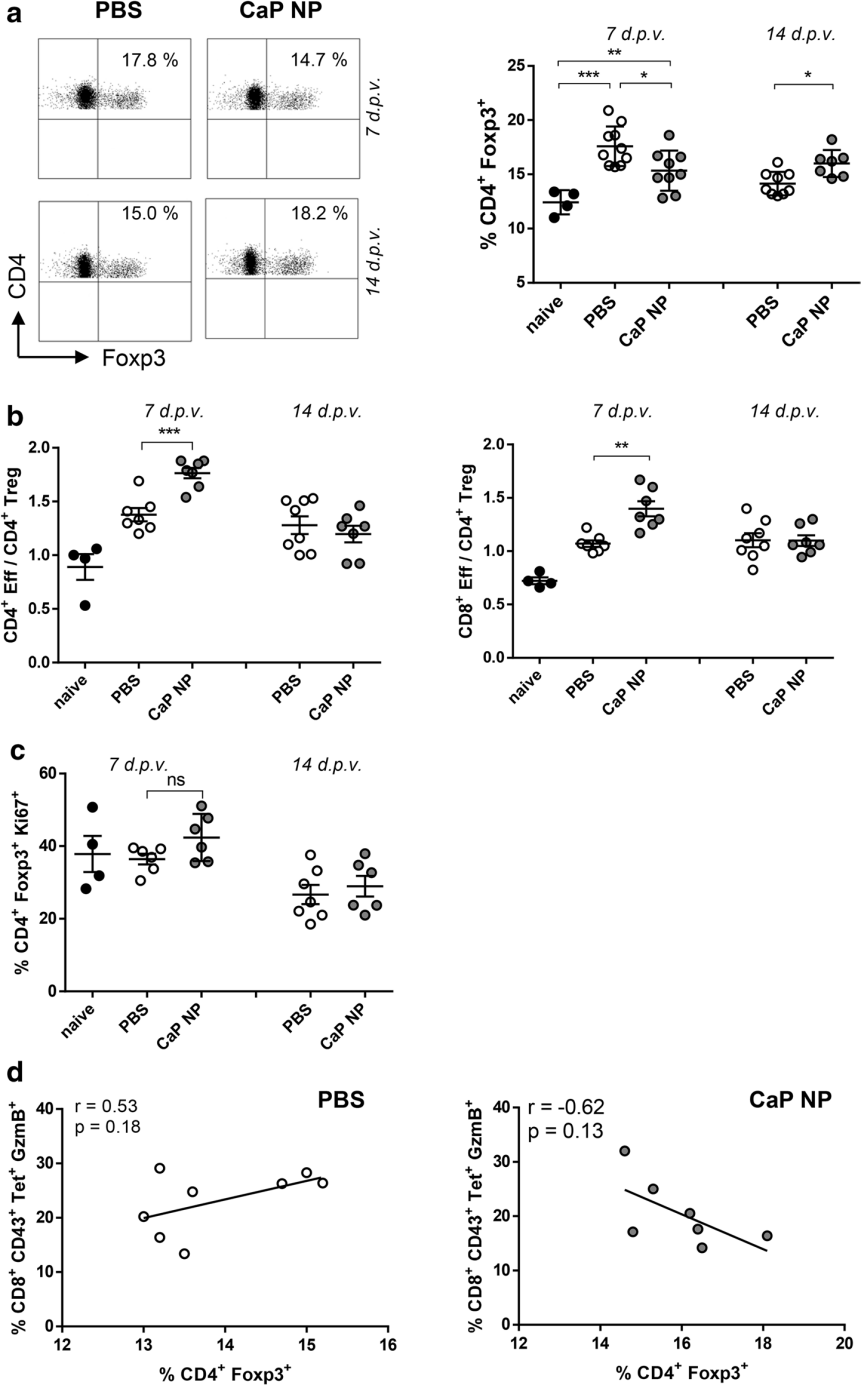
Foxp3^+^ CD4^+^ regulatory T cells down-regulate T cell responses after CaP nanoparticle vaccination. **a** Frequencies of Foxp3^+^ CD4^+^ regulatory T cells were determined by flow cytometry in naïve and chronically FV-infected mice 7 and 14 days post vaccination (d.p.v.) with functionalized CaP nanoparticles. Representative *dot plots* of the analysis are shown. **b** Ratios of fully activated CD4^+^ CD43^+^ or CD8^+^ CD43^+^ effector T cells (EFF) to Foxp3^+^ CD4^+^ T cells. **c** Frequencies of Ki67^+^ Foxp3^+^ CD4^+^ regulatory T cells. **d** Correlation of the percentage of GzmB expressing tetramer stained FV-specific CD8^+^ T cells to frequencies of Foxp3^+^ CD4^+^ T cells 14 d.p.v. with PBS (*left*) or functionalized CaP NPs (*right*). Combined results of three independent experiments are shown. *Bars* represent mean ± SEM. Statistical analysis was performed by student’s *t* test. *p < 0.05; **p < 0.01; ***p < 0.001

We recently demonstrated that therapeutic vaccination with CpG and FV-peptide functionalized CaP nanoparticles decreases the proportion of immunosuppressive CD4^+^ regulatory T cells 7 days after therapeutic vaccination [17]. Nonetheless, it was so far unclear if CD4^+^ regulatory T cells counteract an efficient effector T cells response after therapeutic vaccination. To analyze this issue, FV-resistant C57BL/6 mice were infected with a high dose of FV to induce chronic infection. 6 weeks after infection, mice were therapeutically vaccinated subcutaneously with CpG/gp70/GagL functionalized CaP nanoparticles (Additional file 1: Figure S1B). Splenocytes were isolated from naïve, chronically infected (PBS), and therapeutically vaccinated mice (CaP nanoparticles) 7 and 14 days after treatment, and the percentage of CD4^+^ regulatory T cells was determined. As expected, the proportion of CD4^+^ regulatory T cells was decreased 7 days after vaccination with CaP nanoparticles compared to PBS treated mice. However, we noticed that 14 days after vaccination this reduction was converted into an increase of CD4^+^ Foxp3^+^ regulatory T cells compared to PBS treated chronically infected mice (Fig. 1a). To understand this effect we particularly analyzed the ratio between effector and regulatory T cells at these respective time points. Interestingly, we found that the ratio of fully activated CD43^+^ CD4^+^ and CD43^+^ CD8^+^ effector T cells over CD4^+^ Foxp3^+^ regulatory T cells was significantly elevated 7 days after CaP nanoparticle vaccination compared to PBS treatment; this effect was abolished 14 days after vaccination (Fig. 1b). To check whether the increased regulatory T cell frequency on day 14 after vaccination was the result of cell proliferation we stained for the proliferation marker Ki67. Of note, no significant differences were observed (Fig. 1c). As regulatory T cells are known to control virus-specific CD8^+^ T cell responses during chronic FV infection [5, 6, 25], we analyzed the correlation between CD8^+^ effector T cells and regulatory T cells during vaccination. Importantly, we were able to identify a strong negative correlation between the percentage of FV-specific CD8^+^ CD43^+^ GzmB^+^ T cells and percentage of CD4^+^ Foxp3^+^ T cells in the spleen 14 days after vaccination with CaP nanoparticles. The higher the percentage of CD4^+^ Foxp3^+^ regulatory T cells in the spleen the lower was the percentage of GzmB^+^ FV-specific CD8^+^ T cells (Fig. 1d). Interestingly, this kind of correlation was not seen in the PBS treated control mice (Fig. 1d) nor at day 7 after vaccination which could be due to the change in the T effector-Treg ratios (data not shown). These results clearly demonstrate that although CD4^+^ and CD8^+^ effector T cells expand after therapeutic one shot-vaccination with functionalized CaP nanoparticles, regulatory T cells still have a strong impact on virus-specific immunity.

### Transient ablation of regulatory T cells strongly enhances the therapeutic effect of functionalized CaP nanoparticles

**Fig. 2:**
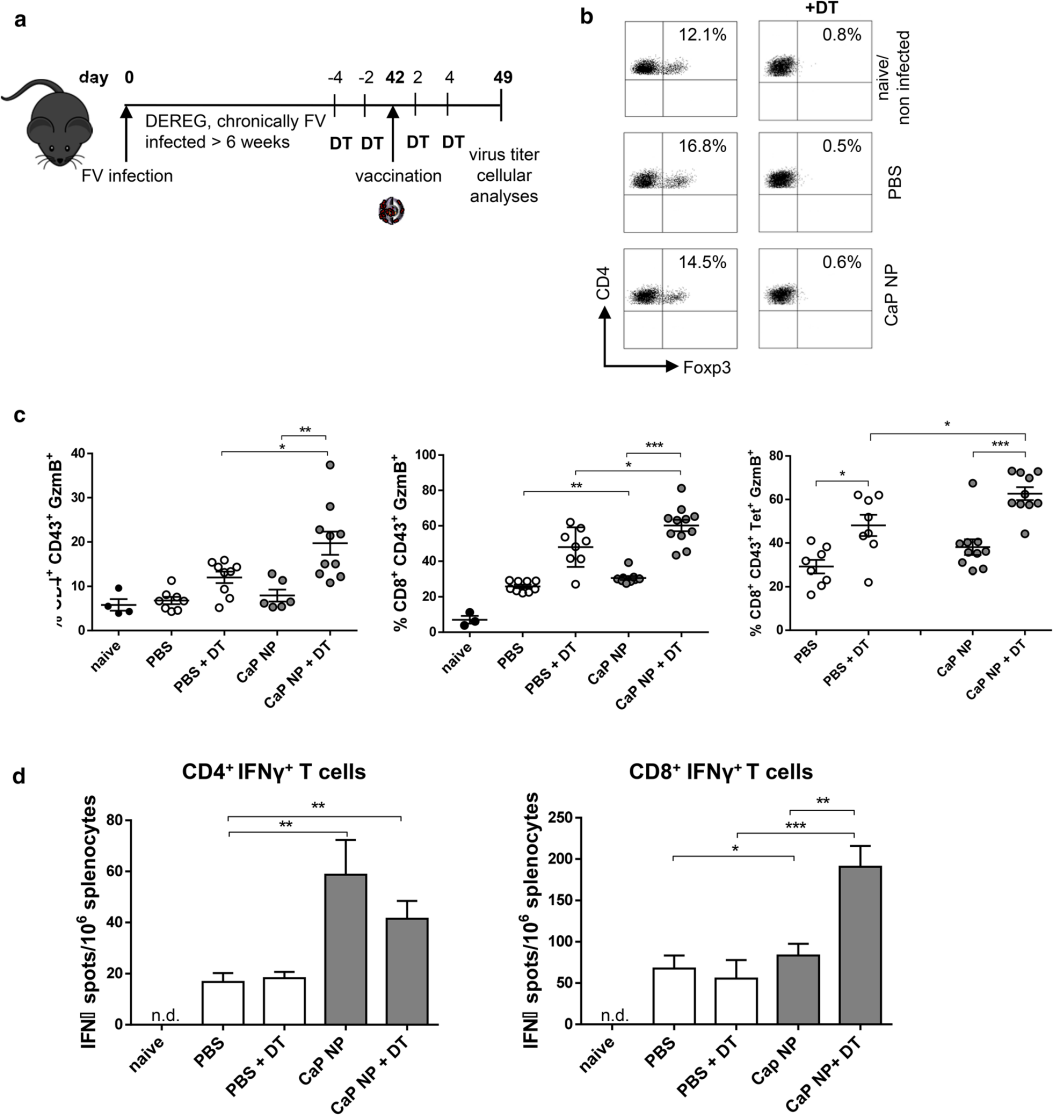
Transient ablation of regulatory T cells strongly enhances the therapeutic effect of functionalized CaP nanoparticles. **a** DEREG mice on C57BL/6 background were vaccinated as described in Additional file 1: Figure S1. In addition, mice were injected with 800 ng/mouse of diphtheria toxin (DT) 2 and 4 days before vaccination as well as 2 and 4 days after vaccination with functionalized CaP nanoparticles. **b** Frequencies of Foxp3^+^ CD4^+^ regulatory T cells and **c** GzmB expression by CD8^+^ or CD4^+^ CD43^+^ T cells in the spleen of naïve and FV chronically infected DEREG mice after vaccination 7 d.p.v. Representative dot plots are shown. **d** Splenocytes were isolated 7 d.p.v. from chronically FV infected DEREG mice treated with or without DT and restimulated ex vivo with 5 µg/ml of GagL_85–93_ or gp70_123–141_ peptide. After 24 h, the numbers of IFN-γ-producing CD4^+^ and CD8^+^ T cells were determined by ELISpot. The figure illustrates the results of three independent experiments. 9 mice per group are shown. *Bars* represent mean ± SEM. One-way ANOVA followed by Bonferoni’s multiple comparison test was performed to analyze statistics of multiple group sets. *p < 0.05; **p < 0.01; ***p < 0.001

**Fig. 3:**
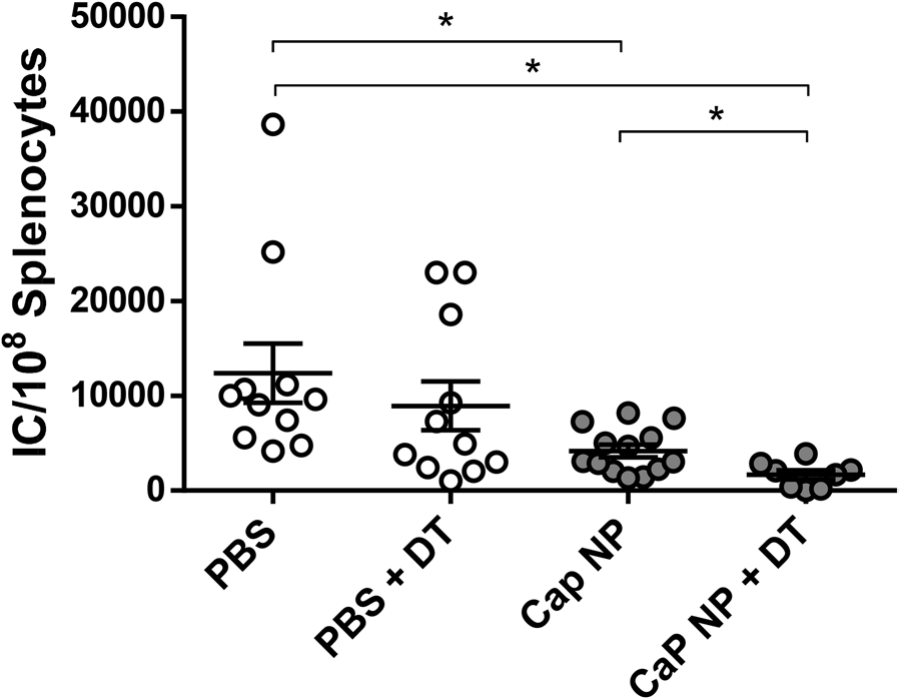
Combinational therapy of regulatory T cell depletion and vaccination with functionalized CaP nanoparticles strongly enhances retroviral clearance. Chronically FV-infected DEREG mice were vaccinated as described in Fig. 2. 7 d.p.v., mice were sacrificed, and infectious centers in the spleen were determined. Results of four independent experiments were combined. *Bars* represent mean ± SEM. One-way ANOVA followed by Bonferoni’s multiple comparison test was performed to analyze statistics of multiple group sets. *p < 0.05

Our results underline the powerful regulatory capacity of Foxp3^+^ T cells on CD8^+^ effector cells with cytotoxic potential during therapeutic vaccination of chronically FV-infected mice. To investigate whether ablation of regulatory T cells further improves the efficacy of functionalized CaP nanoparticles for therapeutic vaccination, we investigated chronically FV infected DEREG (DEpletion of REGulatory T cells) mice in which regulatory T cells can be selectively ablated by the injection of diphtheria toxin (DT) [26]. To ensure that the absence of Tregs will enhance cytotoxicity as well as T cell priming we decided to inject DEREG mice with DT 4 and 2 days before and 2 and 4 days after the vaccination with CpG/gp70/GagL-functionalized CaP nanoparticles at week 6 post FV infection (Fig. 2a), resulting in an up to 99 % depletion of Foxp3^+^ regulatory T cells in naïve, PBS, and CaP nanoparticle treated mice until day 7 post vaccination (Fig. 2b). On day 7 after vaccination, splenocytes were isolated and analyzed for the expression of GzmB by CD4^+^ and CD8^+^ effector T cells. Interestingly, the strongest increase in the frequency of activated cytotoxic CD4^+^ CD43^+^ GzmB^+^ and CD8^+^ CD43^+^ GzmB^+^ T cells was observed after the combination of CaP nanoparticle vaccination and depletion of regulatory T cells. Although the depletion of Tregs led also to an increase in gag-specific cytotoxic CD8^+^ T cells, the combination of CaP nanoparticle vaccination and Treg ablation even stronger increased the amount of those cells compared to DT treatment alone or CaP nanoparticle vaccination alone (Fig. 2c). In addition, ex vivo restimulation of splenocytes with the GagL peptide revealed that the depletion of regulatory T cells during vaccination further augmented the number of IFN-γ producing virus-specific CD8^+^ T cells compared to DT treatment or nanoparticle vaccination alone (Fig. 2d). The application of DT alone was not sufficient to significantly decrease the viral loads in mice. One shot-immunization with CpG/gp70/GagL-functionalized CaP nanoparticles already reduced viral loads in the spleen, and this effect was further enhanced by depletion of regulatory T cells during the immunization process (Fig. 3). These data clearly demonstrate that affecting the number of regulatory T cells before therapeutic vaccination could be crucial to effectively treat chronic viral infection.

### Antagonizing chemokine receptor 4 on regulatory T cells does not improve the anti-viral effect of therapeutic vaccination with functionalized CaP nanoparticles

Depletion of regulatory T cells in humans, as performed in the transgenic DEREG mouse model, is still elusive. Regulatory T cell depletion by anti-CD25 monoclonal antibodies can lead to enhanced immunity; however, a CD25-targeted approach is associated with several drawbacks. First, regulatory T cell depletion might lead to appearance of autoimmune manifestations, as reported in experimental animals. Secondly, activated effector CD4^+^ and CD8^+^ T cells also express CD25 and could also be eliminated by CD25 depletion [27]. Therefore, alternative approaches, focusing on inhibition instead of depletion of regulatory T cells might be of high interest for upcoming therapies.

**Fig. 4:**
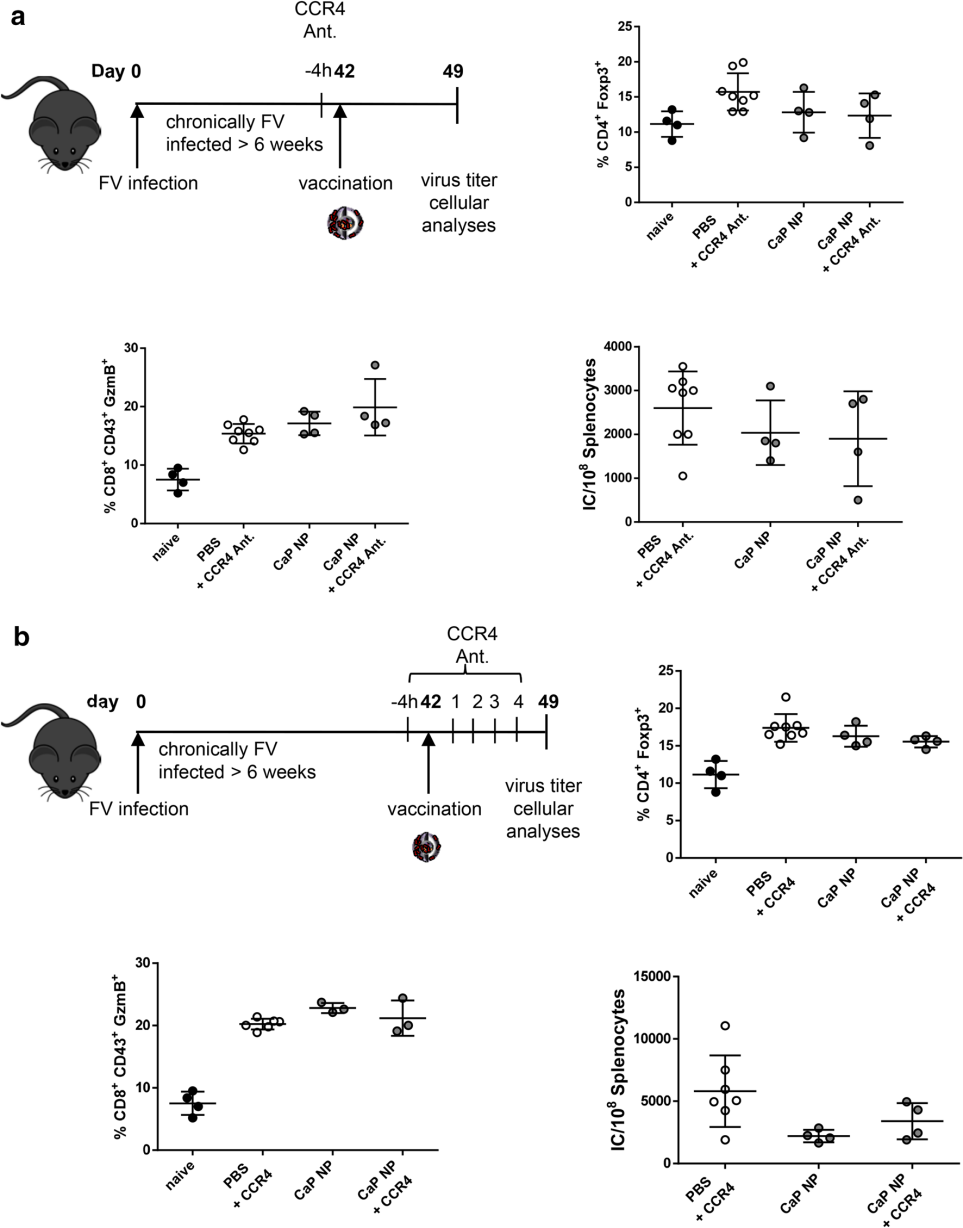
CCR4 antagonist treatment does not enhance vaccination efficacy. C57BL/6 mice were chronically infected with FV (>6 weeks) and therapeutically vaccinated either with PBS or functionalized CaP nanoparticles. In addition mice were injected with 2.5 µg CCR4 antagonist 4 h prior to vaccination (**a**) or in addition on day 1, 2, 3, and 4 post vaccination (**b**). 7 days later mice were sacrificed and analyzed for the percentages of Foxp3^+^ CD4^+^ T cells, CD43^+^ granzyme B expressing CD8^+^ T cells in the spleen are depicted. Infectious centers indicating the viral load in the spleen 7 days after vaccination in combination with 2.5 µg CCR4 antagonist 4 h prior or also on day 1, 2, 3 and 4 post vaccination (**b**) are shown. Results of one experiment are shown. *Bars* represent mean ± SEM. One-way ANOVA followed by Bonferoni’s multiple comparison test was performed to analyze statistics of multiple group sets

Blockade of chemokine receptor 4 (CCR4) by an antagonist was recently reported to inhibit migration of regulatory T cells to tumors, and thereby enhancing anti-tumor vaccination [28, 29]. Human and murine regulatory T cells, but not naïve or effector T cells express CCR4, and the ligands for CCR4, CCL22 and CCL17, are mainly secreted by activated DCs and macrophages. So far, it has not been tested if the combination of anti-viral vaccination with the blockade of CCR4 has a potentiating effect on the immune response. Therefore, we vaccinated chronically FV infected mice with functionalized CaP nanoparticles or with a combination of CaP nanoparticles and an antagonist for CCR4, respectively. 7 days after vaccination T cell responses and viral loads were determined. Pretreatment of mice with a CCR4 antagonist 4 h before nanoparticle vaccination did not modulate the frequencies of regulatory T cells in the spleen compared to nanoparticle vaccination alone nor did the combinational treatment enhance the CTL response (Fig. 4a). In consequence, no differences in the viral loads were detectable between vaccination with nanoparticles and the combinational protocol (Fig. 4a). In addition, a prolonged inhibition of regulatory T cell migration by treatment of mice 4 h before and on day 1–4 after the vaccination did also not enhance anti-viral immunity (Fig. 4b). Consequently, treatment of mice with a CCR4 antagonist to inhibit the migration of regulatory T cells to the side of action does not represent an alternative approach for anti-retroviral therapy.

## Discussion

Viruses such as HBV or HIV possess the ability to evade from the immune system by several mechanisms, like viral evolution or exhaustion of effector T cells which can lead to persistent infection. The currently available treatments for many of these chronic infections do not lead to satisfactory results. For example antiretroviral therapy (ART) is able to suppress HIV replication, the most prominent member of the retrovirus family, but the fact that HIV persists in reservoirs prevents HIV cure by ART. Therefore, there is a strong need to develop new strategies for therapeutic vaccination against chronic infection. Nanomaterials are discussed as part of potential immune-based therapeutic treatment to reactivate the host’s immune response [12, 30]. In our latest report, we demonstrated that the application of CpG and viral peptide functionalized CaP nanoparticles leads to significant reactivation of T cell responses and improves virus control in murine chronic FV infection [17]. We also noticed that Tregs have a strong impact on virus-specific immune responses during chronic retrovirus infection [5]. They seem to have a significant effect on the cytotoxicity of CD8^+^ T cells during acute chronic FV infection by inhibiting the production of cytotoxic molecules such as granzyme A and B [25]. Thus, the aim of the current study was to determine whether the combinational therapy of nanoparticle-based vaccination with depletion of Tregs could strongly enhance the cytotoxic T cell (CTL) response and opens new options in the fight against chronic retroviral infection.

Our current study demonstrates that a combination of depletion of immunosuppressive Tregs and therapeutic immunization with functionalized CaP nanoparticles of chronically retrovirus infected mice significantly reduced viral loads by efficiently reactivating the cytotoxic potential of virus-specific CD8^+^ and CD4^+^ effector T cells compared to therapeutic vaccination alone. It therefore underlines the considerable influence of Tregs on the effector T cell response during immunotherapy which should be considered for the development of new vaccination strategies.

Tregs are a subset of CD4^+^ T lymphocytes with the ability to down-regulate the immune system [31]. They are the key modulators of the establishment and/or maintenance of viral chronicity and constitute a barrier to efficient vaccination and immunotherapeutic strategies [32]. The implication of regulatory T cells in chronic viral infection was first described in mice infected with FV [33, 34] and was then extended to other persistent viruses, including HIV [35], HBV [36], and HCV [2]. Especially for HIV patients, it was shown that Tregs similarly to the situation in chronic FV infection accumulate in lymphoid tissues and suppress the anti-HIV response [35, 37]. Thus, it is becoming increasingly clear that controlling this immunosuppressive cell subset would have widespread clinical applications to fight life-threatening viral diseases [38]. In addition, our current study shows evidence that the Treg population also counteracts the effects of immunotherapeutic vaccination which implies that controlling this subset could further enhance immunotherapy. Suvas et al. [39] have shown that treatment of mice with anti-CD25 antibodies to impair the CD25^high^ expressing Treg population resulted in enhanced antigen-specific CD8^+^ T-cell responses accompanied by reduced viral burden in herpes simplex virus-1 infected mice. In addition, we could demonstrate in recent studies that ablation of Tregs from mCMV- and FV-infected mice by using DEREG (DEpletion of Treg) mice was associated with elevated anti-viral CD8^+^ T-cell responses showing a restored functionality [5, 40]. Also human studies have highlighted the effects of Tregs in suppressing antiviral effector responses. Macatangay et al. [41] showed that reduced frequencies of virus-specific Tregs during vaccination are important for an efficient therapeutic anti-HIV vaccine since this enables the broad activation of effector T cells. Furthermore, an decrease HIV-specific Treg response was associated with an effective DC-vaccine induced immunity in HIV-infected patients [42].

However, the depletion of Tregs should be done with caution since it is known that long term depletion of Tregs can lead to severe autoimmunity syndromes [43]. Temporary Treg depletion or the addition of a Treg blocker along with the vaccine should be considered. Particularly, transient inhibition of Treg migration by using small molecule antagonists to CCR4 was shown to provide robust antigen-specific CD8^+^ T cell responses during anti-tumor vaccination [28, 29]. However, treatment of chronically FV-infected mice with a CCR4 antagonist before and/or during vaccination with functionalized CaP nanoparticles within our experimental setting did not improve the vaccination success. Therefore, further studies are needed to elucidate how to manipulate Treg numbers or function during anti-viral vaccination.

Several lines of evidence suggest now that the depletion of Tregs during therapeutic immunization of chronically retrovirus infected mice improves the vaccination efficacy of functionalized CaP nanoparticles. First, depletion of Tregs during immunization enhanced the relative number of virus-specific IFN-γ producing CD8^+^ T cells. Second, the strongest increase in the frequency of total activated virus-specific cytotoxic CD8^+^ CD43^+^ GzmB^+^ T cells was observed after a combined depletion of Tregs and immunization. Since granzyme B has been associated with virus control and killing of virus infected cells in both FV and HIV infection [44, 45], induction of cytotoxic T cells is crucial for a therapeutic vaccine. Finally, depletion of Tregs during the therapeutic immunization of chronically FV-infected mice resulted in the lowest viral loads compared to all other treatments tested in this study. Thus Tregs clearly control the CTL response induced by therapeutic vaccination and may reduce its effect.

## Conclusion

Our findings underline that a combination of Treg ablation and therapeutic nanoparticle-based vaccination confers robust antiviral immunity and a sustained control of chronic virus infection.

## Methods

### Mice

C57BL/6 mice were purchased from Harlan Laboratories (Harlan Winkelmann GmbH, Borchen, Germany). DEREG (DEpletion of REGulatory T cells) mice (expressing eGFP and diphtheria toxin receptor under the control of the forkhead box P3 [Foxp3] promoter) on C57BL/6 background were described previously [26]. All mice used in the experiments were 8–10 weeks old at time point of infection.

### Cells and cell culture

A murine fibroblast cell line from Mus dunni was maintained in Roswell Park Memorial Institute (RPMI) medium containing 10 % FCS and 50 µg mL^−1^ penicillin/streptomycin. Cell lines were maintained in a humidified 5 % CO_2_ atmosphere at 37 °C.

### Preparation of functionalized nanoparticles

CpG and viral peptide functionalized multi shell CaP nanoparticles were prepared as described previously [17]. Briefly, nanoparticles were prepared by fast mixing of aqueous solutions of calcium nitrate (6.25 mM) and diammonium hydrogen phosphate (3.74 mM). Immediately after mixing, 1 mL of the calcium phosphate nanoparticle dispersion was mixed with 0.2 mL CpG (63 µM = 400 µg mL^−1^) and 0.05 mL of the viral peptides GagL_85–93_ and gp70_123–141_ (1 mg mL^−1^) were added to form an outer shell of biomolecules Then, calcium nitrate solution (0.5 mL), diammonium phosphate solution (0.5 mL) and CpG solution (0.2 mL, 63 µM) were subsequently added to form further layers of calcium phosphate and CpG. This led to triple-shell nanoparticles with the composition CaP/CpG + peptide/CaP/CpG (from core to shell). The final concentrations of CpG and GagL/gp70 peptide in the nanoparticle dispersion were 65.3 µg mL^−1^ (or 10.3 µM) and 20.4 µg mL^−1^, respectively. All inorganic salts were of p.a. (pro analysi) quality. The particles were characterized by scanning electron microscopy (SEM) and dynamic light scattering in the same way as described [17].

### TLR-ligand, viral peptides and CCR4 antagonist

The phosphorothioate-modified class B CpG 1826 was purchased from Eurofins MWG Operon (Ebersberg, Germany). The FV protein derived Gag and gp70 peptide sequences containing MHC I and MHC II-restricted epitopes were synthesized with the following sequences: GagL_85–93_, CCLCLTVFL; gp70_123–141_, EPLTSLTPRCNTAWNRLKL (JPT Peptide Technologies GmbH, Berlin Germany). The CCR4 antagonist CAS 864289-85-0 was purchased from Merck Millipore (Darmstadt, Germany).

### Antibodies and flow cytometry

The monoclonal antibodies αCD4 (clone RM4-5), αCD8 (clone 53–6.7) and αCD43 (clone 1B11) were obtained from BD Biosciences Pharmingen (Heidelberg, Germany). Monoclonal anti-human allophycocyanin (APC)-conjugated αGzmB antibody (clone GB12, Invitrogen, Karlsruhe, Germany) was used for intracellular granzyme B staining. The αFoxp3 antibody (clone FJK-16s) was purchased from ebioscience (Frankfurt, Germany). Intracellular staining with αFoxp3 and αGzmB was performed as described previously [17]. To detect FV-specific CD8^+^ T cells a PE-conjugated recombinant MHC-class I H2-D^b^ tetramer (Beckman Coulter) presenting the FV GagL85-93 peptide was used. Data was acquired by using an LSR II instrument using DIVA software (BD Biosciences).

### Friend virus and chronic infection

A lactate dehydrogenase-elevating virus (LDV) containing B-cell-tropic, polycythemia-inducing Friend virus-complex (FV) was obtained from BALB/c mouse spleen cell homogenate 14 days post infection. To induce chronic FV infection, naïve FV-resistant C57BL/6 mice were infected with 15,000 spleen focus forming units (SFFU).

### Vaccination of mice

For anti-viral therapeutic vaccination of chronically FV infected and vaccination of naïve mice for in vivo DC activation studies mice were immunized subcutaneously with 100 µL PBS or CaP nanoparticles functionalized with CpG/GagL/gp70 (10.3 µM CpG and 40.8 µg mL^−1^ GagL/gp70 peptide; CaP/CpG/GagL/gp70/CaP/CpG) in both hind footpads (50 µL each) 6 weeks post FV infection as described before [17].

### Depletion of Foxp3^+^ regulatory T cells

To deplete Foxp3^+^ regulatory T cells, DEREG mice were injected intraperitoneally with diphtheria toxin (DT) (Merck, Germany) diluted in PBS. 800 ng in total per mouse was injected 4 and 2 days before as well as 2 and 4 days after vaccination with PBS or functionalized CaP NPs.

### IFN-γ ELISpot assay

Interferon-γ-producing cells were evaluated by ELISpot with a mouse ELISPOTPLUS kit (MABTECH AB, Nacka Strand, Sweden). For evaluation of Interferon-γ-producing cells after therapeutic vaccination of chronically infected mice, spleen cells or lymph node cells from a cell pool of brachial, axillary, inguinal, and popliteal lymph nodes were restimulated. Restimulation of 2.5 × 10^5^ cells was done with 5 µg mL^−1^ GagL_85–93_ or gp70_123–141_ peptide in a 96-well ELISPOT plate for 24 h. IFN-γ-spots were stained according to the manufacturer’s instructions. After the plate was dried, the number of spots was counted by an AID ELISPOT reader using ELISPOT 6.0 software (Mabtech, Nacka Strand, Sweden).

### Infectious center assay

Determination of viral loads by infectious center assay was performed as described previously [46]. Spleens were rinsed with RPMI containing 10 % FCS and 50 µg mL^−1^ penicillin/streptomycin. Spleen cells were counted and serially diluted before seeding them onto *Mus dunni* cells and incubated under standard tissue culture conditions for 3 days, fixed with ethanol and labeled with the primary F-MuLV Env-specific MAb 720. After washing, a secondary horseradish peroxidase (HRP)-conjugated rabbit antimouse Ig antibody (Dako, Hambrug, Germany) was added. Foci representing infectious centers were detectable after adding of aminoethylcarbazole (Sigma-Aldrich, Deisenhofen, Germany) as substrate for HRP. Foci were counted and infectious centers (IC)/spleen values were calculated.

### Statistical analysis

Statistical analysis was performed by using student’s *t* test or One-way ANOVA to compare multiple groups using Bonferroni’s multiple comparison test. Data analysis was performed using Prism 6.0 software (GraphPad, La Jolla, CA). Statistical significance was set at the level of *p* < 0.05.

### Ethics statement

Animal experiments were performed in strict accordance with institutional, state, and federal guidelines. The protocol was approved by the North Rhine-Westphalia State Agency for Nature, Environment and Consumer Protection (LANUF) (Permit Number: G1433/14). All efforts were made to minimize suffering.

## Additional file

10.1186/s12977-016-0258-9 A) Schematic illustration of functionalized multi shell calcium phosphate (CaP) nanoparticles. B) C57BL/6 mice were chronically infected with FV (>6 weeks) and therapeutically vaccinated either with PBS or functionalized CaP nanoparticles. 7 or 14 days post vaccination (d.p.v.), mice were sacrificed for analyzes. C) The percentage of CD4^+^ CD43^+^ and CD8^+^ CD43^+^ effector cells is shown. D) Frequency of granzyme B (GzmB) expressing CD43^+^ CD8^+^ T cells and CD43^+^ CD8^+^ FV-specific T cells identified by tetramer staining recognizing the H-2D^b^-restricted FV gag epitope is depicted. E) 7 and 14 d.p.v., mice were sacrificed, and infectious centers in the spleen were determined. The figure summarizes the results of 3 independent experiments. Statistical analysis was performed by student’s *t*-test. **p*<0.05; ***p*<0.005.
