# Supplementary material for: Combination of nanoparticle-based therapeutic vaccination and transient ablation of regulatory T cells enhances anti-viral immunity during chronic retroviral infection
Source: Retrovirology. 2016 Apr 14;13:24. doi: 10.1186/s12977-016-0258-9 (PMC4831142; doi:10.1186/s12977-016-0258-9)
Supplement: Supplementary file 1 — 10.1186/s12977-016-0258-9 A) Schematic illustration of functionalized multi shell calcium phosphate (CaP) nanoparticles. B) C57BL/6 mice were chronically infected with FV (>6 weeks) and therapeutically vaccinated either with PBS or functionalized CaP nanoparticles. 7 or 14 days post vaccination (d.p.v.), mice were sacrificed for analyzes. C) The percentage of CD4+ CD43+ and CD8+ CD43+ effector cells is shown. D) Frequency of granzyme B (GzmB) expressing CD43+ CD8+ T cells and CD43+ CD8+ FV-specific T cells identified by tetramer staining recognizing the H-2Db-restricted FV gag epitope is depicted. E) 7 and 14 d.p.v., mice were sacrificed, and infectious centers in the spleen were determined. The figure summarizes the results of 3 independent experiments. Statistical analysis was performed by student’s t-test. *p<0.05; **p<0.005. [file 12977_2016_258_MOESM1_ESM.pptx]

## Slide 1
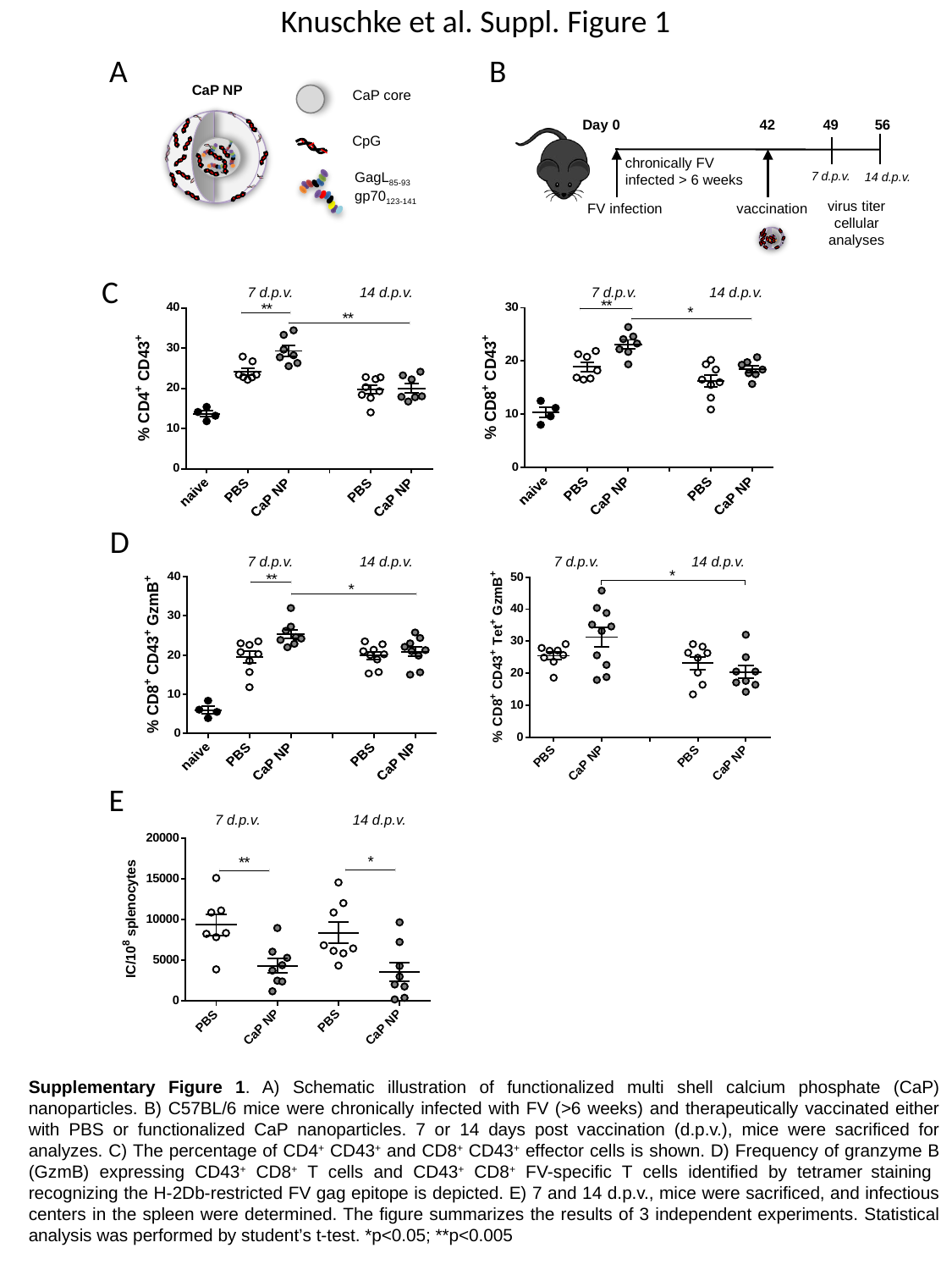

Knuschke et al. Suppl. Figure 1
A
B
CaP NP
CaP core
Day 0
42
49
56
chronically FV
infected > 6 weeks
7 d.p.v.
14 d.p.v.
virus titer
cellular analyses
FV infection
vaccination
CpG
GagL85-93
gp70123-141
C
7 d.p.v.
14 d.p.v.
7 d.p.v.
14 d.p.v.
D
7 d.p.v.
14 d.p.v.
7 d.p.v.
14 d.p.v.
E
7 d.p.v.
14 d.p.v.
Supplementary Figure 1. A) Schematic illustration of functionalized multi shell calcium phosphate (CaP) nanoparticles. B) C57BL/6 mice were chronically infected with FV (>6 weeks) and therapeutically vaccinated either with PBS or functionalized CaP nanoparticles. 7 or 14 days post vaccination (d.p.v.), mice were sacrificed for analyzes. C) The percentage of CD4+ CD43+ and CD8+ CD43+ effector cells is shown. D) Frequency of granzyme B (GzmB) expressing CD43+ CD8+ T cells and CD43+ CD8+ FV-specific T cells identified by tetramer staining recognizing the H-2Db-restricted FV gag epitope is depicted. E) 7 and 14 d.p.v., mice were sacrificed, and infectious centers in the spleen were determined. The figure summarizes the results of 3 independent experiments. Statistical analysis was performed by student’s t-test. *p<0.05; **p<0.005
